# Provision of HIV services to psychiatric inpatients in Botswana: Challenges and recommendations

**DOI:** 10.4102/sajpsychiatry.v29i0.1990

**Published:** 2023-02-28

**Authors:** Maria A. Qambayot, Sarita Naidoo

**Affiliations:** 1 Discipline of Public Health Medicine, School of Nursing and Public Health, University of KwaZulu-Natal, Durban, South Africa

**Keywords:** HIV services, treatment, psychiatric inpatients, healthcare providers, recommendations, challenges

## Abstract

**Background:**

The high prevalence of HIV among psychiatric inpatients is well-documented, yet little is known about the provision of HIV services for these patients.

**Aim:**

This qualitative study aimed to explore and understand healthcare providers’ challenges with providing HIV services to psychiatric inpatients.

**Setting:**

This study was conducted at the national psychiatric referral hospital in Botswana.

**Methods:**

The authors conducted in-depth interviews with 25 healthcare providers serving HIV-positive psychiatric inpatients. Data analysis was performed using a thematic analysis approach.

**Results:**

Healthcare providers reported challenges with transporting patients to access off-site HIV services, longer waiting periods for antiretroviral therapy (ART) initiation, patient confidentiality, fragmented services for treatment of comorbidities, and a lack of patient data integration between the national psychiatric referral hospital and other facilities such as the Infectious Diseases Care Clinic (IDCC) at the nearby district hospital. Providers’ recommendations for addressing these challenges included the establishment of an IDCC at the national psychiatric referral hospital, connecting the psychiatric facility to the patient data management system to ensure integration of patient data, and provision of HIV-related in-service training to nurses.

**Conclusion:**

Psychiatric healthcare providers advocated for on-site integration of care for psychiatric illness and HIV among inpatients to address the challenges of ART provision.

**Contribution:**

The findings suggest the need to improve the provision of HIV services in the psychiatric hospitals in order to ensure better outcomes for this often-overlooked population. These findings are useful in improving clinical practice for HIV in psychiatric settings.

## Introduction

The bidirectional and multifaceted relationship between human immunodeficiency virus (HIV) and psychiatric disorders has been well-established.^1,2,3,4,5,6^ Previous studies have reported that individuals with severe mental illness often engage in HIV-risk behaviours such as substance abuse, sharing drug injecting needles and unsafe sexual activities,^7,8,9,10,11,12^ thereby increasing their risk of HIV acquisition. Furthermore, people living with HIV are more likely to be at increased risk of developing mental disorders because of neurological damage caused by the HIV virus, side effects of antiretroviral therapy (ART) or HIV-related stigma and stress.^13,14^

Several studies in various sub-Saharan settings have reported a high prevalence of HIV among individuals with mental illness, ranging from 11.3% to 50%.^2,15,16,17^ A HIV prevalence of 31% was reported among psychiatric inpatients in Botswana.^18^ Access to people-centred and holistic testing, treatment, care and support services is essential for the well-being of people living with and vulnerable to HIV.^19^ Basic services such as HIV screening have been recommended to be offered routinely and be mandatory.^20,21^ Unfortunately, most psychiatric settings are not well-equipped to provide healthcare services for both HIV and psychiatric disorders in an integrated manner. Unintegrated care for HIV in psychiatric patients may result in inconvenience, unnecessary expenses, and clinicians’ delayed or uninformed decisions, ultimately contributing to poor clinical outcomes.^22^

There is an increased call for better integration of mental health and HIV services.^5,6,23,24,25^ An effectively integrated programme for HIV and psychiatric disorders is recommended to be sensitive to collaboration, coordination and communication across multidisciplines.^26^ Several studies have reported various challenges to integrate HIV and mental healthcare.^27,28^ Among the challenges are inadequate human and financial resources, dearth of data or evidence-based practices, double burden of stigma, structural challenges and a lack of training on HIV services among psychiatric health providers.^27,28,29,30^ Despite these challenges, there is evidence of successful integration of HIV and psychiatric services. Currently, in Rwanda, psychiatric patients receive routine screening and counselling for HIV and within-facility initiation for ART for those testing positive.^31^ In addition, in South Africa, a psychiatric facility effectively integrated on-site HIV services from screening to ART provision, with staff trained as ART providers.^32^ They also integrated ART and psychiatric medication into a single pharmacy with a shared database.

Although there is high prevalence of HIV in psychiatric inpatients, there is a paucity of published studies describing the provision of HIV services to psychiatric inpatients. This study, therefore, sought to describe and explore healthcare providers’ challenges and recommendations regarding the provision of HIV treatment and care to psychiatric inpatients at the national psychiatric referral hospital in Botswana.

## Research methods and design

### Study design

This was a qualitative exploratory study that examined the healthcare providers’ experiences regarding the provision of HIV services among the psychiatric hospitalized patients. A comprehensive literature review, focusing mainly on provision of HIV services for psychiatric patients, informed the development of the study design, objectives and research questions.

### Study setting

This study was conducted at the 300-bed capacity Sbrana Psychiatric Hospital in Botswana. This national psychiatric referral hospital provides psychiatric care and services to both hospitalized patients and outpatients. According to information obtained from the hospital during this study, an average of 250 patients were admitted per month in 2016. A previous study conducted at this hospital reported an HIV prevalence of 31% among adult patients (with a documented HIV status) admitted between 01 January 2011 and 31 December 2012.^18^ Although the hospital provides highly specialized psychiatric services, comprehensive care for other comorbidities such as HIV is provided through referrals to the nearby district hospitals. Upon admission and with their consent, all patients with unknown HIV status are screened at the facility for HIV using rapid testing. The process of ART initiation commences following the confirmation of a positive result. Inpatients who test negative during their admission are retested after 3 months and immediately following any physical altercations resulting in injury. The HIV-positive inpatients are referred to access HIV services at the Infectious Diseases Care Clinic (IDCC) at the nearby district hospital. Patients whose records show that they have been on ART and have defaulted are referred for reinitiation to the district hospital. Healthcare providers at the district hospital are specialized in providing ART and have the capacity to dispense antiretrovirals (ARVs) and conduct viral load testing services. Nurses at Sbrana Hospital are responsible for the daily administration of ARVs for patients on ART; they also closely monitor antipsychotic and ARV treatment side effects. Doctors at Sbrana Hospital sometimes recommend the change of ARV regimen if they discover adverse side effects.

### Study population

Healthcare providers (medical doctors and nurses) who worked in this facility for 6 months and above and directly involved in providing HIV care to inpatients were invited to participate in this study. Study participants were recruited through purposive sampling.^33^ The potential participants were recruited based on their roles in the wards. After receiving permission from the hospital, the matron assigned one nurse to introduce the researcher to the potential participants. Of the 27 participants initially invited to participate, only two declined without providing reasons.

### Data collection

In-depth interviews (IDIs) using semi-structured interview guides were used to explore healthcare providers’ experiences and views on provision of ART to psychiatric inpatients. The interview guide was designed to gather information about providers’ experiences with ART provision, gaps in HIV-related services for inpatients, and strategies or recommendations to address these gaps. A short questionnaire was also administered to collect demographic data. Interviews were conducted from January to February 2020 and were completed within 20 days. All interviews were conducted in English, lasted between 30 min and 45 min and were carried out in a private room at the hospital. All interviews were conducted by the researcher (lead author). A research assistant, experienced in qualitative methodology, was present at all interviews and served as the notetaker. The lead author and research assistant are not healthcare providers and are not employed at the hospital where the data were collected. The interviews were audio-recorded, with participant’s consent and subsequently transcribed. Detailed notes were captured in instances where participant consent was not granted for audio recordings.

### Data analysis

Interviews were transcribed verbatim and analysed manually using an inductive thematic analysis approach.^34^ This process was guided by Braun and Clarke’s six-phase framework^34^, which involves familiarisation with the data, generation of initial codes, searching for themes, reviewing themes, defining and naming themes, and final analysis and write-up of the report. The researcher and research assistant read all transcripts, agreed on the coding process and thereafter analysed the data independently. They met frequently during the data analysis stage to verify and review the outcome of the coding and to identify and refine key themes. This process was undertaken to ensure trustworthiness of the study findings.

### Ethical considerations

This study was approved by the University of Kwazulu-Natal Biomedical Research Ethics Committee (ref: BE346/19) and the Ministry of Health and Wellness in Botswana (HRDC #000870). Written informed consent was obtained from all participants prior to any data collection. Data were stored electronically, password-protected and accessed only by researchers. The study was done in a hospital where the number of medical doctors was small therefore limited identifiers are given to protect the anonymity of the participants.

## Results

A total of 25 healthcare providers, 5 medical doctors and 20 nurses, participated in the interviews. Twelve of the healthcare providers were female. As discussed here, healthcare providers identified several key challenges linked to the unavailability of comprehensive on-site HIV treatment and care services. They also shared recommendations to improve provision of HIV services in this setting.

### Challenges

#### Transporting patients to access off-site HIV services

Currently, all inpatients on ART are transported to the IDCC at the nearby district hospital to access their ARVs, refills, and undergo quarterly reviews and viral load assessment. Patients with good adherence and viral load suppression are often given refills for 3 months. Nurses accompany the patients during these visits. Most of the nurses interviewed expressed their apprehension, while accompanying these patients to the IDCC.

Nurses highlighted the stressful and risky situations they are placed in while transporting patients to the district hospital:

‘To nurses taking patients to [*the district hospital*] with a psychiatric patient who have unpredictable behavior is a challenge that nurses go through every day. A patient may relapse while out there and harass others, no one will be willing to assist. As nurses this is a stressful part of our work that no one else knows.’ (nurse)

They also reported that the situation is worse when accompanying patients who are unhappy about their hospitalisation:

‘We suffer stress us nurses of moving around with our patients who totally rely on us for support. I also think that these patients can pose a potential harm because some want to go home and they regard this as a prison. So going out with one of them and I am just alone, I always worry if we will come back safely.’ (nurse)

Some doctors also echoed this concern:

‘It is somehow risky for a nurse to go with a psychiatric patient, may be a forensic one, to IDCC for reviews. These ones tend to be very calm but they can do anything so I assume the work of taking these patients IDCC must be traumatic to our nurses.’ (doctor)

Furthermore, providers highlighted that the hospital wards were understaffed when nurses are off-site accompanying inpatients to the district hospital. This was reported as significant challenge among the providers:

‘We are already short staffed and therefore it is risky for only one nurse remaining here in the ward.’ (nurse)

#### Longer waiting periods for antiretroviral therapy initiation

Providers explained that the IDCC at the district hospital only operates on four working days from Monday to Thursday for external patients. So, if a psychiatric patient is admitted to Sbrana Hospital on Thursday and tests HIV positive, this patient has to wait until Monday to be initiated on ART. A similar scenario is applicable to those patients who are ready to be reinitiated and to those who are admitted and require an ARV refill urgently:

‘It is not right for the patient who is ready for initiation to wait until we send them to another facility for them to access their treatment and normally we have Monday to Thursday if a patient test positive on Thursday and they are ready to be initiated they have to wait until Monday for them to get their initiated.’ (nurse)

It was reported that reinitiation may be delayed by up to a week if adequate documentation is not available such as the type of ARVs the patient was using. This often happens when patients are admitted unaccompanied by a family member and without their ART treatment cards. The healthcare providers have to search for this information from family members or from the facilities these patients were referred from:

‘For those who are brought without their pill, without treatment card and or unaccompanied by family member we track the previous care giver, and this may take about 5 days and if we cannot locate the care givers we work with [the district hospital] IDCC to track in their records the medication that the patient was using.’ (nurse)

In addition to being transported to the district hospital IDCC, patients are also required to join the queues and wait for their turn to be assisted:

‘Delays are another challenge. When a patient has to be assisted outside our facility they have to travel, join the queue, which will not be the case if they have to be treated here.’ (nurse)

#### Patient confidentiality

Providers raised issues about patients’ concerns regarding confidentiality of their HIV status:

‘If a patient is tested here, instead of receiving treatment here we refer to another institution and this at times causes friction between us and patients. [*They*] say that you told us about confidentiality and now you are referring me to someone else in another institution. Even if we tell them about shared confidentiality, they really don’t like it.’ (nurse)

#### Fragmented health service delivery

Despite a significant number of patients suffering from two or more comorbid health conditions, there is no holistic care available for these patients at the psychiatric hospital:

‘We need to give holistic treatment, but we don’t do that here. Almost 40% of patients have at least one mental illness and one physical condition. Some even have two or three physical conditions. You can imagine we have to refer them to other hospitals or local clinics for them to get treated for these conditions.’ (nurse)

#### Lack of patient data integration

Providers highlighted that they were not able to access the patients’ HIV-related information from the database used by the IDCC at the district hospital. The psychiatric facility was not connected to the integrated patient data management system (IPMS), which is used across other health facilities in the area:

‘We have a problem of not accessing these patients HIV treatment information in our database. Our hospital is not linked to IDCC database system and there is always a hassle to get access to vital HIV treatment information for our patients, especially when they have defaulted.’ (doctor)

### Recommendations to improve HIV treatment and care

#### Establishment of an HIV Infectious Diseases Control Center

Most of the nurses who were interviewed indicated the urgent need for an IDCC to be established at Sbrana Hospital. They further explained that unutilized spaces at the hospital could be used for this purpose. They suggested that nurses at the hospital who are already trained on HIV treatment provision could be assigned to work at the IDCC, if established. This would facilitate ART provision and address many of the current challenges experienced:

‘As a hospital we need IDCC here. Some nurses have been trained as prescribers. They can be pooled to serve at this centre. We don’t even need to hire new personnel. We also have plenty of space; we can have one room or two in these unutilized buildings around the hospital. This will reduce delays for re-initiation and initiation and reduce issues we face when we take these patients for reviews.’ (nurse)

Some doctors also emphasized the importance of establishing an integrated treatment facility at the hospital:

‘As a medical doctor I wish our patients receive comprehensive treatment. Having patients moving from one facility to another indicates that the services are not integrated. This is a referral facility, maybe we need to expand our services to having units that care for physical conditions associated to psychiatric illness. For example, our clients may be having other comorbidities such as HIV, TB [*Tuberculosis*], hypertension, diabetes etc. Those physical conditions commonly attached to psychiatric illness may be attended here if we pool resources to make that happen.’ (doctor)

Other medical doctors stated that their hospital is specialized in psychiatric conditions, and provision of ART services on-site would require additional human resources. They did, however, recommend that ARV refills could be performed from their pharmacy:

‘Maybe in terms of refills, rather than going out of the facility for refills, our pharmacy should be equipped to give then the quick refill but we are specialized referral hospital I don’t think we need to give all types of services because that will require more resources.’ (doctor)

### Patient data integration

Providers proposed that the hospital should be connected to the IPMS system so that they would be able to update information and data related to the inpatients:

‘There is a need to integrate the hospital to IPMS that is used by other health facilities so that there are no gaps in patient information. As for now our data is not in IPMS and not connected with other facilities.’ (doctor)

### The HIV-related in-service training and workshops

Some nurses indicated that when they joined Sbrana Hospital they were not provided with any HIV-related training, as the hospital is a specialized facility focusing on psychiatric illnesses only:

‘We are not trained on HIV/AIDS [*acquired immunodeficiency syndrome*] care and treatment and even if there is a slot for training at the Ministry of Health our staff here are not given that opportunity because our services are specialized to psychiatric conditions.’ (nurse)

The respondents mentioned that they would like to receive in-service trainings and workshops related to HIV:

‘We are not really capacitated to give any specialized care to the HIV positive patients. We come here to care for psychiatric patients, and any other problem we have to refer patients to the specialized settings out there.’ (nurse)

## Discussion

The results of this study demonstrated that while basic HIV services such as HIV testing, counselling and administration of ARVs are provided in this study setting, there is an undeniable need for the availability of more comprehensive on-site HIV care and better integration of services for other comorbidities. Healthcare providers reported challenges with transporting patients to access off-site HIV services, patient confidentiality, fragmented services for treatment of comorbidities, longer waiting periods for ART initiation, and a lack of patient data integration between the study setting and other facilities such as the IDCC at the nearby district hospital.

Previous literature has shown that working in psychiatric facilities is both challenging and stressful.^35,36,37^ The present study revealed the additional stress and apprehension that nurses experienced while accompanying patients to access off-site HIV services. Off-site access to ART services was also associated with longer waiting time for ART initiation. Given the lack of literature on providers’ views of accessing ART services off-site, this is an important information to be considered when developing strategies to improve referral processes, while considering the well-being of both patients and providers. It may therefore be beneficial for future studies to investigate the impact of off-site referrals for care and treatment of psychiatric inpatients.

Patients in this setting who were initiated on ART at a different facility complained about issues around confidentiality. These complaints may have been raised because of fear of disclosure and double stigma.^38^ While confidentiality is an ethical obligation and the right of patients, it is important that during initial consultation with patients, psychiatrists should explain that there is ‘shared confidentiality’ among healthcare providers specifically when it involves referrals for further treatment or care.^39,40^ Patients will then understand the necessity of information sharing among healthcare providers while respecting and maintaining their confidentiality.^41^ In the interest of the patient’s health, non-consensual disclosure of their HIV status may be necessary. According to the *Botswana Public Health Act (2013)*, patients’ HIV status can be disclosed to other healthcare providers involved in the care of the patient.^42^ Thus, patients’ education should cover the importance of referrals and assure patients that providers in all settings have obligations to keep patients’ information confidential.

In this study, a lack of integration and ART initiation capacity on-site was linked to longer waiting periods for ART initiation.^29^ In addition, delayed ART initiation for inpatients may have been because of logistics of patients being accompanied to an external facility.^43^ Fragmented services for treatment of other comorbidities was also a challenge. Similar reports in other settings indicated that separate treatment of the two conditions resulted in increased care costs and poor health outcomes.^44,45,46^ In this study setting, there was also no well-coordinated integrated system where psychiatric service providers and HIV service providers could readily access patients’ information from either setting. Patients’ records for HIV and psychiatric disorders were not integrated. The ART data captured electronically at the nearby facility where patients received their HIV treatment were not accessible by psychiatric clinicians and vice versa, making it impossible to link patient data across settings. It is important to integrate patient data to allow for treatment continuity.^47^ A compatible electronic patients’ record system across facilities will be effective in allowing healthcare providers access to records detailing the mental health services and physical health services from different facilities.^30^ The integration of patient data for psychiatrics, HIV and other comorbidities will ensure easy access to patient data across facilities, thereby enabling healthcare providers to address patients’ healthcare needs more efficiently. This concurs with reports that, in the United Kingdom, there was no consensus among clinicians regarding how physical healthcare among psychiatric patients should be provided.^43^ Thus, there is a need to develop stronger linkages and referral systems that encourage bidirectional communication between psychiatric health providers and HIV service providers.^41^

To address these challenges, the respondents strongly recommended the introduction of an IDCC at the study site, where all ART services can be offered, as in other primary healthcare facilities in Botswana. This recommendation is aligned to the current move by the Ministry of Health and Wellness to abolish IDCCs and integrate the HIV services into primary healthcare services.^48^ The benefit of using an integrated healthcare model is that it facilitates access to major healthcare services in one setting, thereby reducing the burden of seeking healthcare in different facilities, especially for people with complex needs such as HIV and psychiatric disorders.^49^

In-service training of health workers is one of the most used strategies to improve the quality and coverage of HIV/acquire immunodeficiency syndrome (AIDS) services.^50^ However, in this study, the lack of HIV treatment training was reported as a gap, concurring with findings in other settings.^29,51^ The lack of training on HIV treatment among psychiatric professionals was reported to be the barrier to provision of integrated services for the comorbidities in studies in Zimbabwe and the United States.^43,52^ Often healthcare workers in psychiatric settings do not attend workshops or in-service training because of the tight work schedule and shortage of staff.^53^ In Botswana, general healthcare providers are provided with comprehensive, standardized and coordinated training known as ‘Knowledge, Innovation and Training Shall Overcome AIDS’ (KITSO), which covers a wide range of basic to advanced HIV-related topics.^54^ Unfortunately, nurses in psychiatric setting are not included in this training because they are considered specialized in psychiatric patients. According to the HIV commission,^23^ professionals in psychiatric settings need more training related to HIV and special needs of people living with HIV. Consequently, Joore^55^ considers mental health professionals as an untapped resource in mental health settings for addressing both mental health and HIV/AIDS.

In the researcher’s lens, there is a dilemma between the specialized nature of psychiatric referral hospitals and the call for integration with other conditions such as HIV, especially in most resource-limited settings. While integration may be ideal, it is important to consider the resources required, the scope of integration and if similar integration can be performed in general hospitals for consistency. In most developing countries, the mental health needs of psychiatric patients have not been met and any expansion to management and care of physical conditions may overburden these facilities and strain the limited resources. The authors recommend that a cost-benefit analysis be undertaken to better inform the feasibility of integrated care in psychiatric settings.

### Study’s strengths and limitations

This is the first study to assess the challenges faced with the provision of HIV services in this setting from the providers’ perspective and highlighted the need for the integration of comprehensive HIV services in this specialised psychiatric facility. This information may be used by health providers and policymakers to review current practices and develop new strategies to improve HIV treatment and care provision for psychiatric inpatients. This study, however, has a few limitations. The findings are specific to the perspectives of the respondents who participated in the study and may not be generalisable to other settings. The interviews were conducted within the hospital setting and this may have influenced the individual responses.

## Conclusion

This study highlighted the complexity of providing ART to psychiatric inpatients. To meet the needs of psychiatric inpatients with HIV comorbidity and optimize HIV services, there is a need to fully integrate HIV and psychiatric services in the same facility. Recommendations from healthcare providers could help in development of approaches to improve HIV service delivery for inpatients. While a few of these recommendations may require additional resources, some proposed strategies may be adopted with currently available resources to initiate the integration of HIV services into psychiatric inpatient care.
